# White Matter Microstructure Abnormalities in Individuals at High Risk for Psychosis: A Meta-Analysis of Fractional Anisotropic Changes Associated With Transition to Psychosis

**DOI:** 10.1192/bjo.2024.220

**Published:** 2024-08-01

**Authors:** Chukwuma Ntephe, Dhwani Desai, Pedro Laguna, Anthony David, Kate Merritt

**Affiliations:** 1 University College London, London, United Kingdom; 2 South West London and St George's Mental Health NHS Trust, London, United Kingdom; 3 Cardiff University, Cardiff, United Kingdom

## Abstract

**Aims:**

Recent studies have focussed on detecting white matter abnormalities in subjects who transition to psychosis (UHR-T). Research suggests that fractional anisotropy (FA), may be decreased in UHR-T. However, global and regional findings have been inconsistent. By objectively combining data in a meta-analysis, we have investigated white matter alterations associated with transition, by comparing FA in UHR-T with subjects that do not transition (UHR-NT) and healthy volunteers.

**Methods:**

The meta-analysis was registered on PROSPERO (ID: CRD42021265348) and followed Preferred Reporting Items for Systematic Reviews and Meta-Analyses PRISMA guidance. A systematic database search of PUBMED and EMBASE identified reports, which were screened by 2 independent researchers (CN and DD) for inclusion, from inception to 20 July 2021. Discrepancies were decided on consensus with a third researcher (KM). Reference lists of eligible studies were also screened. Authors of screened reports were contacted to provide parametric maps. Coordinate-based meta-analysis was conducted using Seed-based *d*-Mapping software to combine parametric map and coordinate data from reports, using a random-effects model. Quality and risk of bias analysis were conducted using the Newcastle-Ottowa Scale. Heterogeneity and sensitivity analyses were also conducted.

**Results:**

The search strategy identified 889 potential studies, from which 6 met eligibility criteria. A total of 71 UHR-T, 142 UHR-NT and 148 healthy volunteers were included. Weighted-mean decreases in FA were observed in UHR-T compared with: UHR-NT (*d* = −0.99; p < 0.0001; 95% CI −1.43 to −0.55); and healthy volunteers (*d* = −0.91; p = 0.04; 95% CI −1.78 to −0.05). The level of heterogeneity for the former was not significant. For UHR-T, regional FA decreases were observed in areas including the left genu of the corpus callosum (Z-score = −1.76, 204 voxels, p < 0.0001) compared with UHR-NT, while FA increases were most observed in the white matter region adjacent to the left postcentral gyrus (Z-score = 1.64, voxels = 16, p < 0.0001). These findings persisted despite sensitivity analyses.

**Conclusion:**

The findings suggest that white matter alterations, specifically in left frontotemporal tracts, are associated with an increased risk of transition to psychosis. The neurobiological implications of these findings, and their contribution to UHR-T prediction efforts, are explored, as are avenues for further research.

Additional Author: Mr Gunnar Gronlid.^1^

